# Presence and Role of Associations of Cancer Patients and Volunteers in Specialist Breast Centres: An Italian National Survey of Breast Centres Associated with Senonetwork

**DOI:** 10.3390/curroncol30090594

**Published:** 2023-09-04

**Authors:** Rosanna D’Antona, Silvia Deandrea, Elisabetta Sestini, Loredana Pau, Francesca Ferrè, Catia Angiolini, Marina Bortul, Lauro Bucchi, Francesca Caumo, Lucio Fortunato, Livia Giordano, Monica Giordano, Paola Mantellini, Irene Martelli, Giuseppe Melucci, Carlo Naldoni, Eugenio Paci, Gianni Saguatti, Corrado Tinterri, Milena Vainieri, Luigi Cataliotti

**Affiliations:** 1 Europa Donna Italia, 20122 Milan, Italy; rosanna.dantona@europadonna.it (R.D.); elisabetta.sestini@europadonna.it (E.S.); loredana.pau@europadonna.it (L.P.); naldoni.carlo@tiscali.it (C.N.); 2 Environmental Health Unit, Agency for Health Protection, 27100 Pavia, Italy; presidente@gisma.it; 3 Management and Health Laboratory, Institute of Management, Department EMbeDS, Sant’Anna School of Advanced Studies, 56127 Pisa, Italy; francesca.ferre@santannapisa.it (F.F.); irene.martelli@gmail.com (I.M.); milena.vainieri@santannapisa.it (M.V.); 4 SOD Oncologia Della Mammella, Breast Unit, DAI Oncologico, Azienda Ospedaliero-Universitaria Careggi, 50134 Firenze, Italy; angiolinic@aou-careggi.toscana.it; 5 Breast Unit, Division of General Surgery, Azienda Sanitaria Universitaria Giuliano Isontina, Hospital of Cattinara, 34149 Trieste, Italy; m.bortul@fmc.units.it; 6 Emilia-Romagna Cancer Registry, Romagna Cancer Institute, IRCCS Istituto Romagnolo per lo Studio dei Tumori (IRST) Dino Amadori, 47014 Meldola, Italy; 7 Department of Breast Radiology, Veneto Institute of Oncology, IRCCS, 35128 Padova, Italy; francescacaumo@gmail.com; 8 Breast Centre, San Giovanni-Addolorata Hospital, 00184 Rome, Italy; lfortunato@hsangiovanni.roma.it; 9 CPO Piedmont, AOU Cittá della Salute e della Scienza, 10126 Torino, Italy; livia.giordano@cpo.it; 10 Medical Oncology Department, Azienda Socio Sanitaria Territoriale Lariana, 22100 Como, Italy; monica.giordano@asst-lariana.it; 11 Screening Unit, ISPRO—Oncological Network, Prevention and Research Institute, 50139 Firenze, Italy; p.mantellini@ispro.toscana.it; 12 SS Radiologia Senologica, ASL ‘SS. Annunziata’, 74100 Taranto, Italy; g.ppe_melucci@libero.it; 13 Italian Group for Mammography Screening, 50131 Firenze, Italy; paci.eugenio@gmail.com; 14 Senology Unit, Local Health Authority, 40139 Bologna, Italy; gianni.saguatti@ausl.bologna.it; 15 Breast Unit, Humanitas Cancer Centre, Rozzano, 20089 Milan, Italy; corrado.tinterri@cancercenter.humanitas.it; 16 A.P.S. Senonetwork Italia, 50144 Firenze, Italy; luigi.cataliotti@gmail.com

**Keywords:** breast cancer, breast centre, associations, patients, volunteers, survey

## Abstract

This article aims to present the results of a national, cross-sectional, voluntary, online survey on the presence and roles of associations of breast cancer patients and volunteers in Italian specialist breast centres. The survey was developed according to standard methods. The questionnaire was pre-tested by a random sample of three breast centres, loaded onto the SurveyMonkey platform, and piloted by one volunteer breast centre. The breast centre clinical leads were invited to participate via email. A link to the online instrument was provided. No financial incentives were offered. The results were reported using standard descriptive statistics. The response rate was 82/128 (65%). Members of associations were routinely present in 70% Italian breast centres. Breast centres most often reporting their presence were those certified by the European Society of Breast Cancer Specialists. Patient support (reception and information, listening, identification of needs, and psychological support) was the primary area where associations were reported to offer services. The magnitude of this phenomenon warrants a study to investigate the impact of the activities of associations on the quality of life of patients and on the cost–benefit ratio of the service, and the modes of their interactions with the nursing staff and the medical staff.

## 1. Introduction

The most important study conducted so far on the role of associations of cancer patients and volunteers (hereafter briefly referred to as “associations”) in health systems, particularly in patient care and support, showed a great deal of diversity of national models [1]. This derives from differences in organisation and governance of national and local health systems coupled with an even more pronounced heterogeneity of health legislation, which encompasses a variety of laws, regulations, directives and other legislative instruments.

As a consequence, the activities undertaken by associations have evolved along many different directions. First, more and more emphasis is currently being placed on the shared decision-making process, in which clinicians and cancer patients collaborate to make shared medical decisions based both on clinical evidence and patient values and preferences [2]. As a parallel phenomenon, associations have increasingly exerted an influence in important areas like health and research ethics, guideline development and implementation [3], research agenda-setting processes, health policy processes, cancer education and awareness programmes [4], and general cancer care strategies. This trend is expected to be boosted by scientific advances, especially by the introduction of new and diversified options to diagnose, characterise, and treat the disease [5].

Second, many associations—especially in Europe—promote group activities for patients, including, for example, physical activities and provision of information to newly diagnosed patients. For these patients, receiving cancer-related information and support may positively affect treatment adherence, psychological well-being, and quality of life [5,6,7], because involvement in an association represents a valuable means to fulfil many disease-related needs.

Third, associations have gained increasing consideration by health and medical research stakeholders, because they can have a function in several research activities, including, for example, conducting clinical trials [8] and quality-of-life studies, especially of long-term survivors. Some of the most common cancers are poorly—or not at all—represented in this type of literature, and long-term survivors are particularly underrepresented [9]. Associations can provide crucial assistance in identifying and contacting the potential target populations of patients [10,11].

In spite of these multifaceted and important functions, a recent systematic review of the medical literature showed that only 18 studies worldwide—for all cancer sites—have formally addressed the type of initiatives undertaken by associations and other similar organisations, the type of care they offer, and the type of evaluation of the related outcomes [12]. There were major differences between associations with respect to the selection of persons. Interestingly, a personal history of cancer was an inclusion criterion in certain instances and an exclusion criterion in others. Differences also existed as to the training programmes (presence/absence, duration, type, and inclusion/exclusion of medical and emotional aspects) and the type of care and support being offered. The evaluation of outcomes was generally poor. Although patients had positive experiences according to all studies, the experiences of volunteers and of health staff members were evaluated much less frequently and with less favourable results. Very importantly, one-third of volunteer cancer patients interviewed in one study reported that the service brought up distressing thoughts about their own cancer experience.

In Italy, an in-depth analysis of the outcomes of associations’ activities is particularly warranted in one specific setting—that of specialist breast centres. A breast centre (also referred to as a breast unit) is defined as the place that provides all breast care services on a multidisciplinary basis, including genetics and prevention, primary treatment, care of advanced disease, supportive and palliative care, and survivorship care [13,14]. In the last 20 years and longer, strong evidence has been collected that patients treated by specialist teams in these centres have a survival advantage [15]. In 2014, the Italian Ministry of Health issued a directive [16] to the Departments of Health of the regional administrations (responsible for healthcare delivery) requiring the creation of a regional network of breast centres in line with the technical criteria established by the European Society of Breast Cancer Specialists (EUSOMA) [13,14]. The directive gave associations an official role and recommended that they provide well-defined types of support, including patient information on access modalities, identification and advocacy of patient needs, assessment of patient satisfaction, provision of support to manage the illness and to engage in rehabilitation programmes, collaboration with the staff in order to ensure equity of access to services, and participation in healthcare quality assessment initiatives. It must be considered that, in Italy, the members of associations include both persons with a personal history of cancer and unaffected volunteers, with the former being the majority.

Subsequently, the presence of one or more associations was included as a recommendation in the EUSOMA requirements and in the certification of Italian breast centres through BCCert [17], that is, the certification scheme of the EUSOMA.

**Figure 1:**
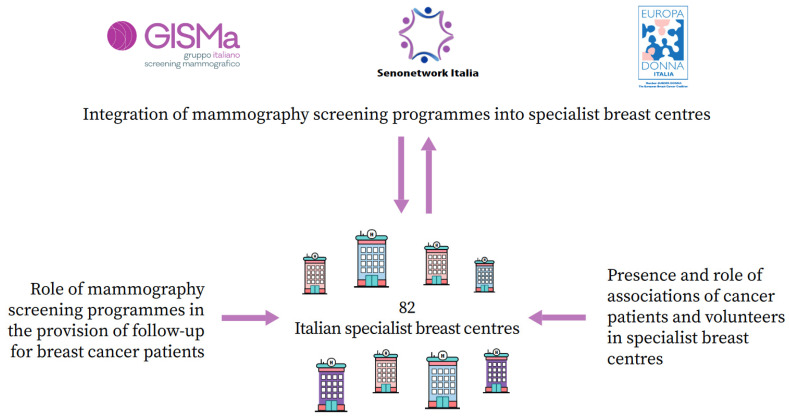
Scheme depicting the three-section design of the national, cross-sectional, voluntary, online survey of Italian specialist breast centres associated with Senonetwork, 2020. The primary objective of the survey was to investigate the integration of mammography screening programmes into breast centres [18]. The secondary objectives were (1) to assess the role of mammography screening programmes in the provision of follow-up for breast cancer patients [19] and (2) to quantify the presence and assess the role of associations of cancer patients and volunteers in breast centres (present study). Of the 128 breast centres associated with Senonetwork in 2020, 82 responded to the survey.

The Italian Group for Mammography Screening (GISMa), Senonetwork, and Europa Donna Italia carried out a national, cross-sectional, voluntary, online survey of Italian specialist breast centres [18,19]. Figure 1 depicts the three-section design of the protocol. The primary objective was to assess the degree of integration of mammography screening programmes into breast centres. One of the secondary objectives was to quantify the presence and assess the role(s) of associations in breast centres. In this article, the survey findings about this topic are briefly presented and discussed.

## 2. Materials and Methods

### 2.1. Survey Development

The survey was developed and implemented according to standard guidelines [20]. The questionnaire addressed appropriate themes proposed by the main national stakeholders. Consideration was given to the relevant national legislation and to the EUSOMA requirements for breast centres [13,14]. The final version of the questionnaire consisted of 73 questions grouped under five domains: (i) breast centre identification and main characteristics, including, among others, the certification according to the EUSOMA standards [17] (questions 1–16); (ii) the breast centre clinical lead’s perception of utility, effort required, acceptability, and facilitating conditions of the integration of the screening programme into the breast centre (17–34); (iii) breast centre’s screening mammogram volume and relationship with the screening programme (35–48); (iv) dimension of integration: organizational (49–51), functional (52–58), service-related (59–64), and clinical (65); and (v) structural and functional details of the integration (65–73).

Four questions concerned the presence and role of associations in breast centres. They were developed by taking into account the 2014 directive from the Ministry of Health. In the directive, the types of support expected from associations were grouped into six major areas: (1) patient reception and information, (2) patient listening and identification of patient needs, (3) psychological support, (4) participation in assessment of the quality of services, (5) participation in fundraising initiatives, and (6) other, including support to engage in rehabilitation programmes, diet sessions, and health groups.

### 2.2. Survey Pre-Testing and Piloting

The questionnaire was pre-tested by a random sample of three breast centres. After collecting their feedback, unclearly worded, ambiguous, and misleading questions were modified. The survey was then loaded onto the SurveyMonkey platform (https://it.surveymonkey.com/, accessed on 31 August 2023) and piloted by one volunteer breast centre. A PDF version of the questionnaire (in Italian) is available on the website of the GISMa [21]. Further details can be found in two previously published articles [18,19].

### 2.3. Survey Process

At the time the survey was conducted, an official national list of breast centres was not available because their creation was still incomplete in some administrative regions. As a consequence, the survey was presented to breast centres associated with Senonetwork, the Italian network of breast cancer services, which is nationally recognised as a representative professional body for breast specialists. The clinical leads of these centres, or the main contact persons, were invited to participate via email. A link to the online instrument was provided. No financial incentives were offered. The survey was conducted between July and October 2020. The emergency caused by the COVID-19 pandemic, particularly severe in breast care services, led to a delay both in the roll-out of the initiative and in the analysis of questionnaire responses.

### 2.4. Data Analysis

In the present article, the results are reported using standard descriptive statistics, that is, frequencies, proportions, medians, ranges, and interquartile ranges (IR).

## 3. Results

### 3.1. Characteristics of Participating Breast Centres

Of the 128 breast centres associated with Senonetwork on 1 July 2020, 82 (65%) responded to the survey. The response rate was higher in northern Italy (53/74 or 72%) than in central–southern Italy (29/54 or 54%). The responding centres had a median of 345 (IR, 250–484) new breast cancer cases per year, and all but one (99%) reported > 150 cases per year. The median number of staff in the multidisciplinary team was 21 (IR, 14–30). The reported median number of mammograms per year was 15,000 (IR, 9000–24,750). Twenty-four (29%) breast centres were EUSOMA-certified through the BCCert scheme. Sixty-one (74%) were formally appointed as breast centres by the regional administrations’ bodies. All of the centres surveyed responded to all questions concerning the presence and role of associations.

### 3.2. Presence and Roles of Associations in Breast Centres

According to the 82 questionnaires filled out, members of one or more associations were routinely present and active in 57 (70%) Italian breast centres. No difference whatsoever in this regard was observed in relation to the size of the centre, as defined on the basis of the number of new breast cancer cases seen per year or the number of health professionals on the multidisciplinary team. The proportion was greater in the north of the country (41/53 or 77%) than in central (10/19 or 53%) and southern Italy (6/10 or 60%).

The subgroups of breast centres most often reporting the presence of associations were those certified through the BCCert scheme (20/24 or 83% versus 37/58 or 64%) and particularly those appointed by a regional administration (54/61 or 88% versus 3/21 or 14%). An official administrative designation as a part of the regional network of breast centres predicted the presence of an association with a considerable degree of sensitivity and specificity.

Table 1 shows the proportion of breast centres where associations had a permanent and active role, by breast centre characteristic and type of support offered. Patient support, that is, reception and information, listening, identification of needs, and psychological support, was the primary area where associations were reported to offer services. In approximately 50% of breast centres, associations were involved in the provision of all of these services. Less often did they participate in healthcare quality assessment initiatives or in providing support to engage in rehabilitation programmes. Interestingly, the participation in fundraising initiatives was as common as patient support initiatives.

## 4. Discussion

### 4.1. Main Findings

With a response rate of about two-thirds, this survey provided sound evidence that members of the associations are routinely active in 70% of Italian breast centres and that breast centres most often reporting their presence are those that are EUSOMA-certified through the BCCert scheme and particularly those officially appointed by a regional administration. Patient reception and information, listening, identification of needs, and psychological support are the main areas of commitment of associations.

### 4.2. Main Comments

The magnitude of engagement of cancer patients and volunteers in favour of women attending the Italian breast centres has probably become much larger than perceived by healthcare professionals and providers. In our opinion, this can be explained by the absence of previous comparable statistics and, possibly, by a general tendency to underestimate the level of commitment of the associations.

We believe that the large presence of officially appointed associations in breast centres and their very frequent engagement in key patient support services indicate the need to conduct a study to determine the qualitative impact of this work. There are two types of endpoints that would be important to measure. First, and most important, it is necessary to formally assess whether and to what extent the efforts made by the associations are capable of improving the quality of life of patients and optimising the cost–benefit ratio of the service. According to Europa Donna Italia, these should be the main objectives of the associations [22]. Ethical and acceptability issues can be overcome using a propensity score model in an observational comparative effectiveness study, with the objective of reducing confounding by covariates that are associated both with the outcome and with the exposure. Propensity score models are increasingly used for observational comparative studies in the area of health services research [23].

Second, other important issues that would need to be better understood are the types and modes of the interactions between the associations and the medical and nursing staff. This is a vital necessity, considering that patient information and support remain the primary areas where associations offer services.

Third, it has also been suggested that the personal experiences of cancer patients and their contact with members of associations should be thoroughly explored [24].

A different but equally interesting aspect to explore is why Italian breast cancer patients and volunteers engage less frequently, on average, in activities that are not directly related to patient support services, such as, for example, interaction with the breast centre staff, in order to ensure equity of access, and participation in healthcare quality evaluation initiatives (both recommended by the 2014 directive of the Italian Ministry of Health) [16]. There is, however, a notable exception: the participation in fundraising initiatives, which was reported almost as frequently as patient support activities. Considering the budget constraints affecting the health system, this interesting finding merits further investigation.

### 4.3. Methodological Considerations

The conduct of the survey was satisfactory both from a technical and a theoretical point of view. Firstly, the SurveyMonkey platform fit well for the purpose and enabled the questionnaire to be created, the responses to be edited and collected, and the results to be analysed in a practical way. Secondly, the highly multidisciplinary composition of the working group allowed us to approach and solve the different issues concerning the development and the management of the survey.

The results of this study must be seen in light of the fact that the representativeness of participating centres is difficult to establish with certainty. When the survey was developed, an official national list of Italian breast centres was not available. We targeted the pool of centres associated with Senonetwork, 128 on 1 July 2020, which represents an acceptable approximation. In this way, we were able to make a reasonable estimate of the response rate. The figure we obtained, 65%, allowed us to rule out a substantial nonresponse bias. Although it is true that participation was lower in central–southern Italy, this simply reflects the fact that the prevalence of active local screening programmes is lower in the south of the country, with approximately 27% of 50–69-year-old women being regularly screened versus 52% in central Italy and 62% in northern Italy [25]. This inevitably caused a lower interest in a survey that addressed primarily the integration between the breast centres and the screening programmes [18]. In any case, the geographic difference in the survey participation rate was not substantial. As a final remark, it should be noted that the number of new breast cancer cases per year was above 150 (minimum acceptable standard according to the EUSOMA criteria) in all of the participating centres except one, which does not reflect a self-selection bias but, rather, an increasing and favourable trend that is ongoing [26].

## 5. Conclusions

This study showed, first, that members of one or more associations of cancer patients and volunteers are routinely present and active in as many as 70% of Italian breast centres and, second, that patient information and support are the main areas where associations offer services. The magnitude of engagement of cancer patients and volunteers in favour of Italian breast centres is probably much larger than perceived by healthcare professionals and providers. Our conclusion is that the impact of this effort on the quality of life of patients and the interactions with the nursing staff and the medical staff need to be formally investigated with an observational comparative effectiveness study.

## Figures and Tables

**Table 1:** Number and (in parentheses) percentage proportion of Italian specialist breast centres where associations of breast cancer patients and volunteers have a permanent and active role, by breast centre characteristic and type of support offered.

Breast Centre Characteristic	Type of Support						Total
	Patient Reception and Information	Patient Listening, Identification of Needs	Psychological Support	Assessment of Quality of Services	Participation in Fundraising Initiatives	Other *	
Total	42 (51)	35 (43)	43 (52)	15 (18)	39 (48)	4 (5)	82
Geographic area							
North	30 (57)	24 (45)	32 (60)	12 (23)	27 (51)	3 (6)	53
Centre	7 (37)	7 (37)	6 (32)	1 (5)	9 (47)	1 (5)	19
South	5 (50)	4 (40)	5 (50)	2 (20)	3 (30)	0 (0)	10
No. of new breast cancer cases per year							
<345	19 (46)	16 (39)	20 (49)	6 (15)	21 (51)	2 (5)	41
≥345	23 (56)	19 (46)	23 (56)	9 (22)	18 (44)	2 (5)	41
No. of staff on the multidisciplinary team							
<21	18 (44)	18 (44)	21 (51)	6 (15)	22 (54)	3 (7)	41
≥21	24 (59)	17 (41)	22 (54)	9 (22)	17 (41)	1 (2)	41
BCCert certification							
No	27 (47)	20 (34)	29 (50)	11 (19)	25 (43)	3 (5)	58
Yes	15 (63)	15 (63)	14 (58)	4 (17)	14 (58)	1 (4)	24
Regional appointment †							
No	2 (10)	0 (0)	2 (10)	0 (0)	2 (10)	0 (0)	21
Yes	40 (66)	35 (57)	41 (67)	15 (25)	37 (61)	4 (7)	61
Hospital classification							
Public hospital	24 (46)	22 (42)	24 (46)	8 (15)	29 (56)	4 (8)	52
Private accredited hospital	2 (40)	2 (40)	4 (80)	1 (20)	3 (60)	0 (0)	5
IRCCS and AOU	8 (57)	4 (29)	8 (57)	4 (29)	3 (21)	0 (0)	14
Private accredited IRCCS	8 (73)	7 (64)	7 (64)	2 (18)	4 (36)	0 (0)	11

BCCert, European Society of Breast Cancer Specialists Breast Centre Certification; IRCCS, Istituto di Ricovero e Cura a Carattere Scientifico (non-university research hospital); AOU, Azienda Ospedaliero-Universitaria (university hospital). * Mainly including patient support to engage in rehabilitation programmes, diet sessions, and health groups. † In Italy, only breast centres receiving an official appointment by the Departments of Health of the regional administrations, which are largely responsible for healthcare provision in the country, become part of the regional network of breast centres.

## Data Availability

The data presented in this article are available from the authors on reasonable request.
